# Simultaneously imaging and quantifying *in vivo* mechanical properties of crystalline lens and cornea using optical coherence elastography with acoustic radiation force excitation

**DOI:** 10.1063/1.5118258

**Published:** 2019-10-08

**Authors:** Yan Li, Jiang Zhu, Jason J. Chen, Junxiao Yu, Zi Jin, Yusi Miao, Andrew W. Browne, Qifa Zhou, Zhongping Chen

**Affiliations:** 1 Beckman Laser Institute, University of California, Irvine, Irvine, California 92612, USA; 2 Department of Biomedical Engineering, University of California, Irvine, Irvine, California 92617, USA; 3 Department of Ophthalmology, School of Medicine, University of California, Irvine, Irvine, California 92617, USA; 4 Gavin Herbert Eye Institute, University of California, Irvine, Irvine, California 92697, USA; 5 Department of Ophthalmology and Biomedical Engineering, University of Southern California, Los Angeles, California 90089, USA; 6 Roski Eye Institute, University of Southern California, Los Angeles, California 90007, USA

## Abstract

The crystalline lens and cornea comprise the eye’s optical system for focusing light in human vision. The changes in biomechanical properties of the lens and cornea are closely associated with common diseases, including presbyopia and cataract. Currently, most *in vivo* elasticity studies of the anterior eye focus on the measurement of the cornea, while lens measurement remains challenging. To better understand the anterior segment of the eye, we developed an optical coherence elastography system utilizing acoustic radiation force excitation to simultaneously assess the elasticities of the crystalline lens and the cornea *in vivo*. A swept light source was integrated into the system to provide an enhanced imaging range that covers both the lens and the cornea. Additionally, the oblique imaging approach combined with orthogonal excitation also improved the image quality. The system was tested through first *ex vivo* and then *in vivo* experiments using a rabbit model. The elasticities of corneal and lens tissue in an excised normal whole-globe and a cold cataract model were measured to reveal that cataractous lenses have a higher Young’s modulus. Simultaneous *in vivo* elasticity measurements of the lens and cornea were performed in a rabbit model to demonstrate the correlations between elasticity and intraocular pressure and between elasticity and age. To the best of our knowledge, we demonstrated the first *in vivo* elasticity of imaging of both the lens and cornea using acoustic radiation force-optical coherence elastography, thereby providing a potential powerful clinical tool to advance ophthalmic research in disorders affecting the lens and the cornea.

## I. INTRODUCTION

**FIG. 1.**
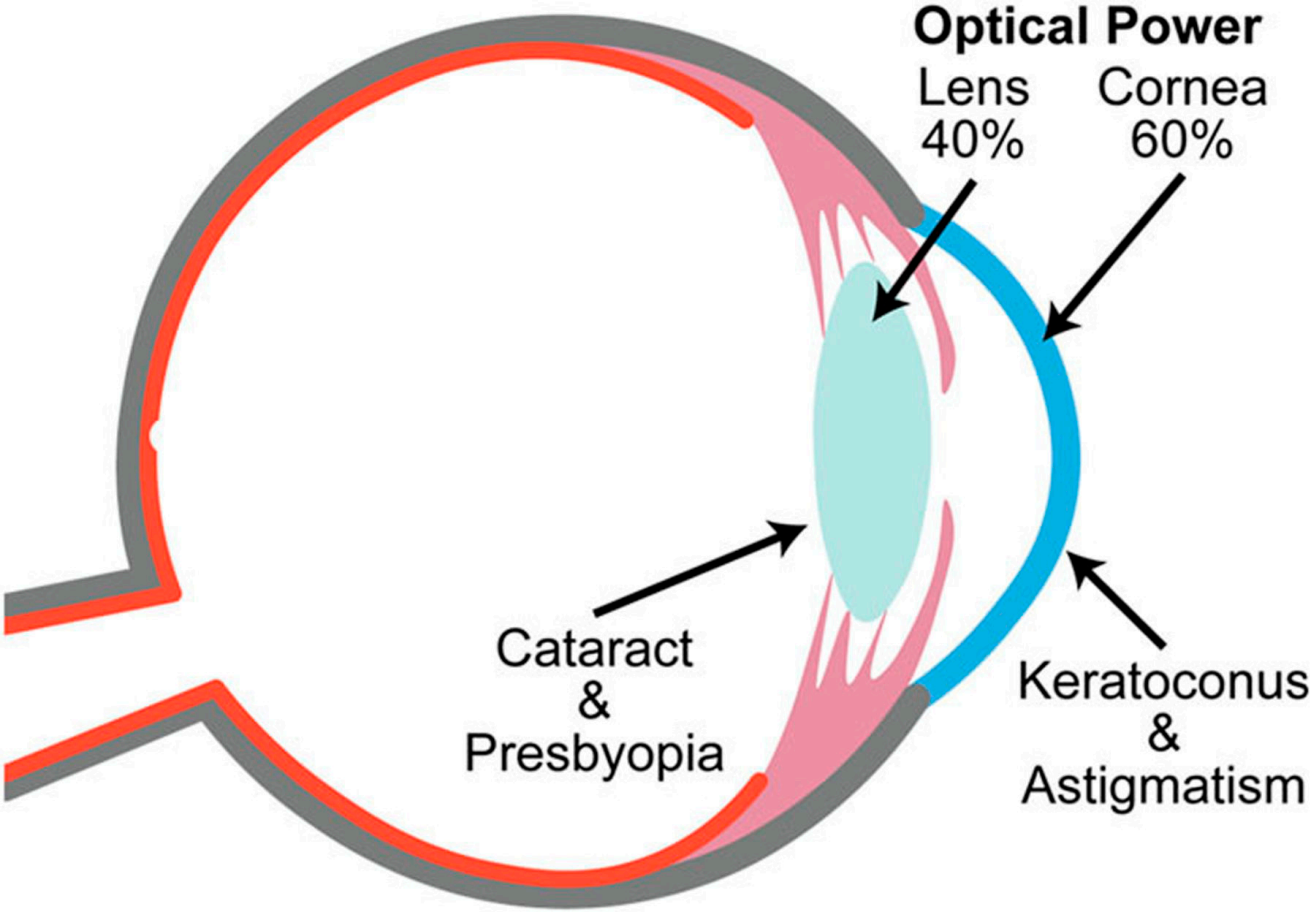
Schematic of the crystalline lens and cornea. The eye optical power provided by the lens is about 40%, and that provided by the cornea is approximately 60%. Common diseases affecting the functionalities of lens and cornea include astigmatism, cataract, keratoconus, and presbyopia.

The eye is a complex organ consisting of several mutually interacting components, with each part bearing biomechanical properties that are closely related to its respective anatomic functionality. Changes in the biomechanical properties are associated with a number of ocular diseases.^1,2^ The cornea and the crystalline lens, like a Keplerian telescope, focus light onto the retina through a series of refractions, allowing us to perceive a sharp image of objects. The light first enters the eye through the cornea, whose curvature contributes approximately 60% of the focusing power of the eye. Thereafter, the biconvex lens provides the remaining refractive power (Fig. 1). In youth, the lens can have its curvature dynamically adjusted to allow adjustment of focal distance through a process known as accommodation.^3^ With age, the lens loses its malleability and its dynamic range of adjustable focus evanesces. With advancing age and in many disease states, the lens becomes more rigid and more opaque and is described as a cataract.^4^ Therefore, the biomechanical properties of the lens are essential to understand the development of refractive disorders. Other disorders, including myopia, hyperopia, and astigmatism, within this optical system may also cause refractive errors.^5–7^ In severe cases, vision quality can be significantly affected, resulting in serious reduction in quality of life.

Contemporary clinical ophthalmic imaging techniques include optical coherence tomography (OCT), Scheimpflug imaging, and confocal microscopy.^8–11^ These techniques enable the study of corneal and lens morphology and clarity. The biomechanical properties of the cornea have been investigated by many research groups^12–17^ because of the superficial location and its ease of accessibility. However, knowledge of lens biomechanics *in vivo* is limited by its less accessible intraocular locations. Therefore, a need exists for a noninvasive approach to study lens biomechanics *in vivo*.

Elastography detects tissue elasticity by visualizing the tissue deformation induced by an external force stimulation as a function of time (i.e., elastic wave propagations).^18^ Several elastography modalities have been developed, including those based on magnetic resonance imaging, ultrasonography, Brillouin microscopy, and optical coherence tomography (OCT), each with its own advantages and limitations.^13,16,19–23^ Magnetic resonance elastography provides a large penetration depth and a wide field of view, but ophthalmology applications are hindered by its low spatial resolution, long imaging time, and cost.^21^ Ultrasound elastography has an improved resolution (~100 *μ*m) but is still insufficient for accurate elasticity evaluation in ocular tissue.^22^ While Brillouin microscopy has also been proposed to investigate the age-related elasticity change in lenses, the correlation between Brillouin shifts and Young’s modulus is still unclear because of the uncertainty of Poisson’s ratio.^23–25^ Optical coherence elastography (OCE), benefiting from the OCT technique, possesses micron-level resolution and a subnanometer axial displacement sensitivity, and it has been applied in ophthalmology to provide quantitative assessment of tissue biomechanical properties with high resolution and sensitivity.^16,26–32^

Mapping and characterizing *in vivo* mechanical properties of the cornea using OCE have been reported by several groups.^12,14,16,33–37^ Our group has recently demonstrated the feasibility of *in vivo* imaging of retina and choroid mechanical properties using an OCE system with acoustic radiation force excitation (ARF-OCE).^28^ Although the lens elasticity has been investigated *ex vivo*, *in vivo* imaging and quantification of lens mechanical properties remain challenging.^38–40^ Furthermore, the cornea and the lens collectively are an optical system, and simultaneous measurements of cornea and lens mechanical properties will yield clinical endpoints for studying tissue biomechanics in health, disease, and treatment.

In this study, we developed an ARF-OCE system to quantify the elasticities of the cornea and the lens. The ocular tissue was excited using an ultrasound transducer, and a swept source OCT system was constructed to detect the shear wave propagations. We first demonstrated the feasibility of ARF-OCE for lens elasticity measurement by comparing *ex vivo* healthy rabbit whole-globes to those with cold cataract. Simultaneous *in vivo* measurements were then performed on both lens and cornea in a rabbit model. Finally, we investigated the *in vivo* elasticity changes in the lens with respect to intraocular pressure (IOP) and age.

## II. MATERIALS AND METHODS

### A. ARF-OCE system setup

A customized phase-sensitive swept-source OCT system was constructed to image the elastic waves of the anterior eye. The laser has a repetition rate of 100 kHz and a center wavelength of 1310 nm. A 90:10 optical fiber coupler splits the output light into the sample and the reference arms, respectively. In the sample arm, the illuminating light propagates to the sample through a circulator, a collimator, and an objective scan lens. A dual-axis Galvo system is incorporated to enable volumetric scanning. In the reference arm, the light propagates through a circulator, a collimator, and a mirror. The backreflected and backscattered light from the reference and the sample arms are collected through the exiting ports of the corresponding circulators into a 50:50 coupler to generate the interference signals, which are then detected by a balanced photodetector. The signal from the detector is digitized using a waveform digitizer (Alazar Technologies, Inc., QC, Canada) and stored in a computer for data processing.

**FIG. 2.**
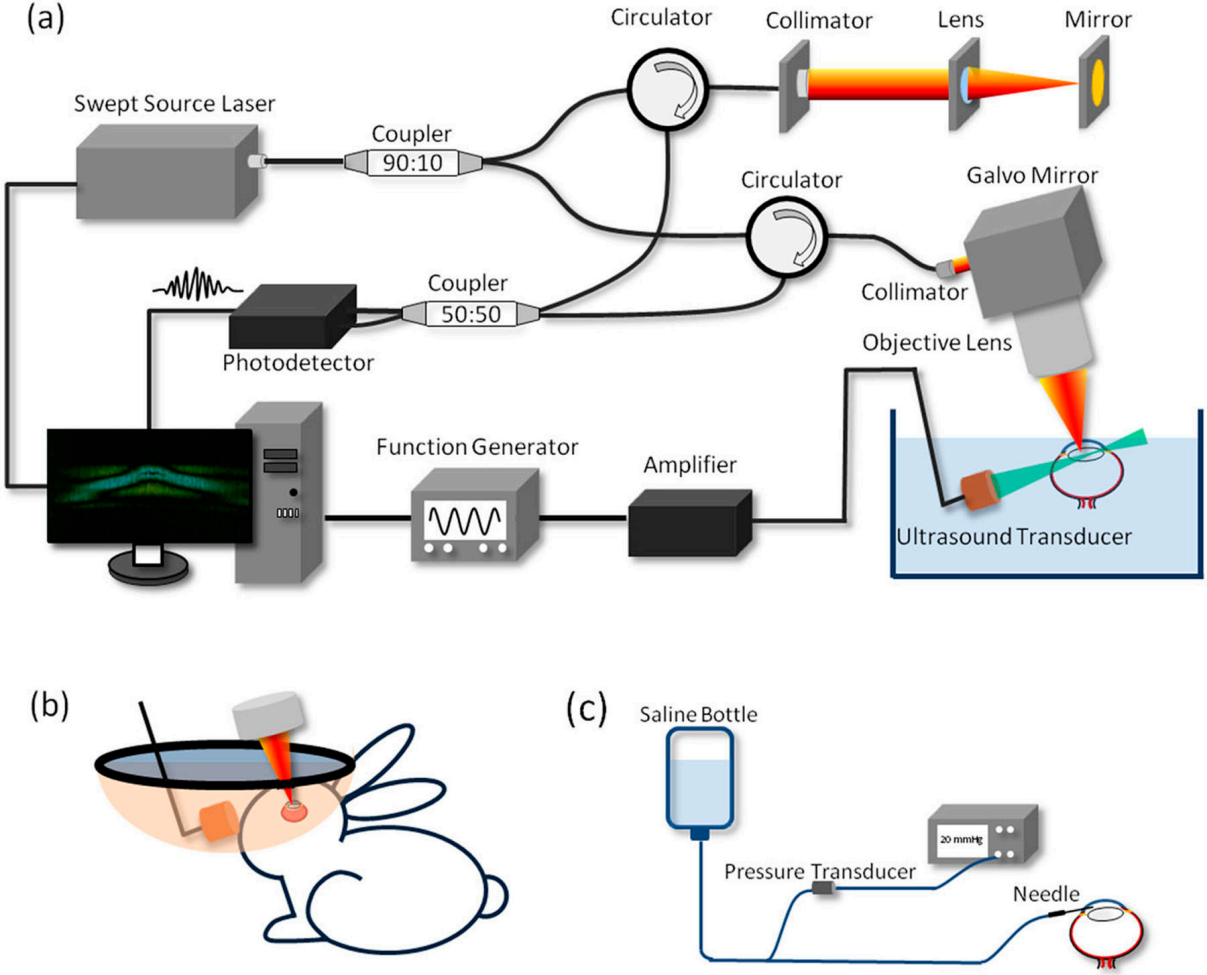
Schematics of (a) the ARF-OCE system, (b) the *in vivo* experiment setup, and (c) the custom-built IOP control system.

To provide the excitation force, a custom-built 4.5 MHz ultrasound transducer with a focal length of 35 mm is incorporated. The transducer is placed approximately orthogonally to the OCT scan lens. The trigger signal from the swept-source laser is utilized to synchronize a function generator to generate a 4.5 MHz sine wave with an amplitude of 800 mV and a duration of 200 *μ*s. The generated wave is amplified by approximately 42 dB and then applied to the focused ultrasound transducer for tissue excitation. The ARF-OCE setup is shown in Fig. 2(a).

For the *in vivo* experiment, the rabbit eye is proptosed within a rubber drape that serves as a container to immerse the eye in sterile phosphate-buffered saline (PBS), as shown in Fig. 2(b). In consideration of the strong optical absorption of water at 1310 nm, the OCT detection beam is rotated by 10° to minimize the required PBS level as well as to avoid a strong reflection from the air-liquid interface, as shown in Fig. 2(b).

The schematic of the custom-built IOP control system is depicted in Fig. 2(c). A sterile saline reservoir is connected to the eye through a 22-gauge needle, and a pressure sensor is integrated to monitor the IOP. Different IOPs of the eye globe can be achieved by adjusting the height of the saline reservoir while monitoring the pressure transducer output.

### B. System synchronization

**FIG. 3.**
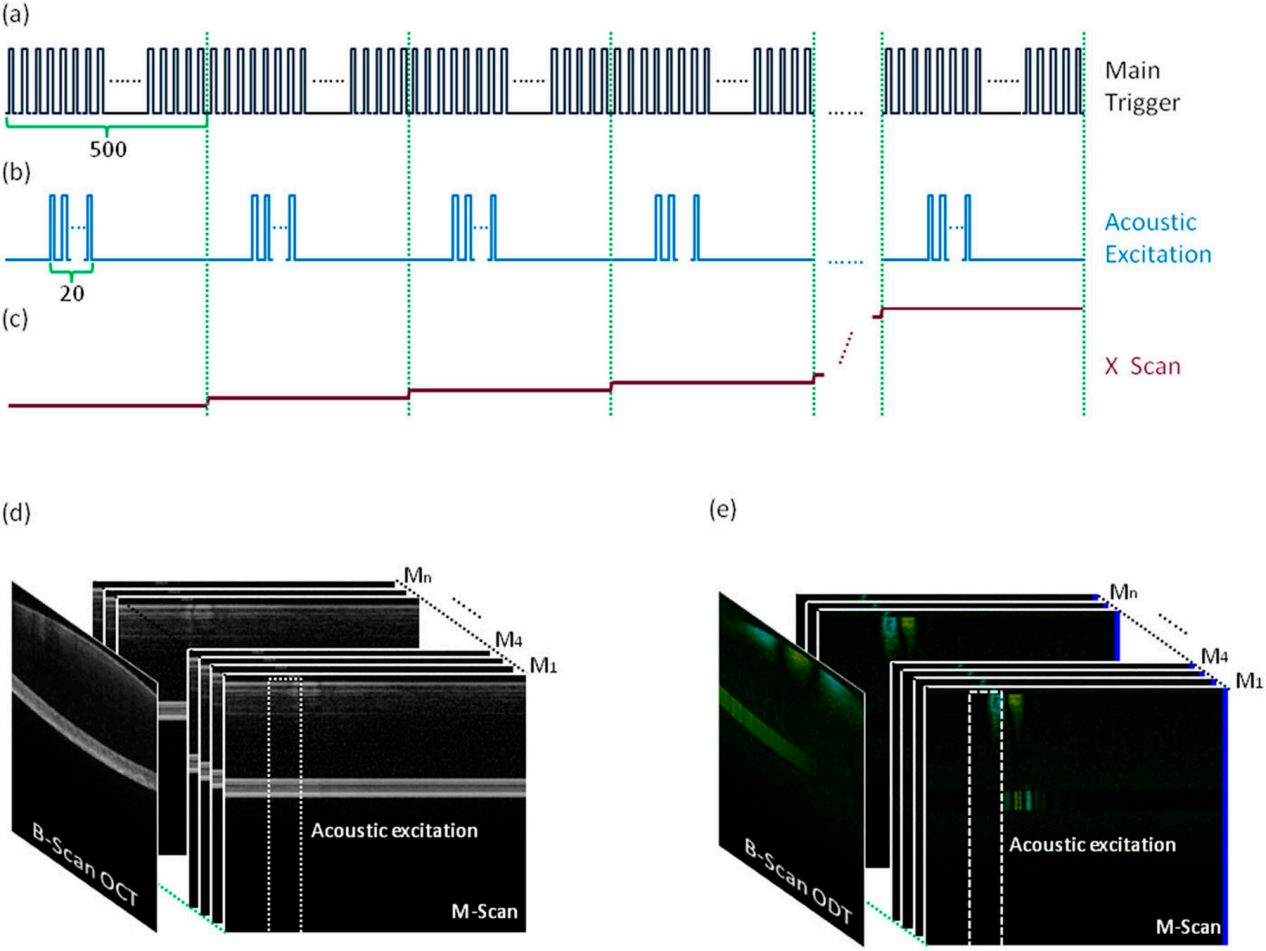
Scanning scheme of the ARF-OCE system. (a) Trigger signals from the laser for synchronization of data acquisition. 500 A-lines consist of one image. (b) Trigger signals for synchronizing the ARF excitation. (c) Signals for the x-axis galvanometer scanner to employ M-B mode scan. (d) OCT image with the M-B mode scan protocol. (e) OCE images with the M-B mode scan protocol.

To visualize the elastic wave propagation in the ocular tissue, an M-B scan protocol is utilized to acquire data. At each position, 500 A-lines are acquired to record the phase change over time (M-mode acquisition). The ultrasound transducer generates the excitation force between the 101st and the 120th A-line scans. After one M-mode data acquisition, the galvanometer scanner moves the detection beam to the next position, and the same M-mode scan is repeated. The scanning protocol is summarized in Fig. 3. The λ trigger signal from the laser is used to synchronize the laser sweeps, data acquisition, ARF excitation, and galvanometers. Using the λ trigger instead of the normal swept trigger provides precise synchronization with the phase of the laser, which is critical for Doppler imaging.

### C. Mechanical characterization

With M-mode OCT images, a temporal phase profile at the lens can be obtained using the following equation:^41,42^

(1) $$\Delta \varphi \;=\;{\text{tan}}^{-1}\frac{\text{Im}\left(F_{m}\times F_{m+1}^{*}\right)}{\text{Re}\left(F_{m}\times F_{m+1}^{*}\right)},$$

where Im() and Re() are the imaginary and real parts of the OCT complex signal, respectively, F_m_ is the complex signal captured at a given position (i.e., M-mode), F_m+1_ is F_m_ at the next time point, and F* is the conjugate complex of F. Then, the temporal phase change, Δφ, can be converted to temporal displacement change, Δd, by the following equation:

(2) $$\Delta d\;=\;\frac{\lambda_{0}}{4\pi n}\Delta \varphi ,$$

where λ_0_ is the center wavelength of the laser source and n is the refractive index. With M-B mode OCT images, Δd as a function of time on the lens could be visualized, and the elastic wave velocity V can be determined by finding the slope of Δd(t).

When the excitation focus is near or on the sample surface, the detected elastic wave in the lens can be considered as a Rayleigh wave. For a homogeneous isotropic sample, Young’s modulus E can be calculated based on the Rayleigh wave velocity V_R_ using the following equation:^43^

(3) $$E\;=\;\frac{2\rho \;\times \;{\left(1\;+\;v\right)}^{3}}{{\left(0.87\;+\;1.12v\right)}^{2}}\;\times \;V_{R}{}^{2},$$

where Poisson’s ratio ν is 0.5, the lens density ρ is 1183 kg/m^3^, and *V*_*R*_ is the detected wave velocity.^44,45^

The elastic wave propagation in the cornea is partly guided by the top and the bottom boundaries of the cornea with consecutive reflections, known as the Lamb wave. In a vacuum, Lamb wave velocity can be calculated using the following equation:^46^

(4) $$V_{L_{-}\text{vacuum}}\;=\;\sqrt{\frac{2\pi \;\times \;f\;\times \;h\;\times \;V_{s}}{\sqrt{3}}},$$

where *f* is the frequency of the Lamb wave, *h* is the cornea thickness, and *V*_*s*_ is the shear wave velocity. Since the cornea is submerged by liquid, the corresponding Lamp wave velocity can be corrected by multiplying a factor of $1/\sqrt{2}$ due to a total leakage of the compressional wave and total reflection of the shear wave at both boundaries $\left(V_{L}=V_{L_{-}\text{vacuum}}/\sqrt{2}\right)$. Given that $E=3\rho \;\times \;V_{s}^{2}$ the corresponding Young’s modulus can be calculated based on the Lamb wave velocity using the following equation:

(5) $$E\;=\;\frac{9\rho \;\times \;V_{L}^{4}}{{\left(\pi \;\times \;f\;\times \;h\right)}^{2}},$$

where the corneal density is 1062 kg/m^3^ and *V*_*L*_ is the detected wave velocity.^47^

### D. *Ex vivo* rabbit experiment preparation

The whole eye globe from a healthy rabbit was obtained within 3 h of euthanasia. A one percent agar was molded around the globe to secure it for imaging. The mounted globe and the ultrasound transducer were submerged in PBS during imaging. After imaging the normal globe, the globe was immersed in iced saline for 15 min to induce a cold cataract for diseased model imaging.

### E. *In vivo* rabbit experiment preparation

For the induction of general anesthesia, the rabbit (male, New Zealand white rabbit) was administrated a ketamine-xylazine mixture (35 mg/kg and 5 mg/kg, respectively) for initial anesthesia. Two drops of proparacaine HCI were applied topically for further local anesthesia. After conforming the proper depth of anesthesia, the rabbit was placed on a stage for position adjustment. Its eye was then carefully proptosed, and a latex drape with an aperture was put through the globe to serve as a container. In the latex drape container, sterile PBS was filled to submerge the eye and the ultrasound transducer. Additional ketamine (17.5 mg/kg) was administered via subcutaneous injection as needed. The IOP control system was used to regulate the IOP of the globe. After imaging, the rabbit was euthanized via intravenous pentobarbital overdose. Five rabbits were imaged in total. All procedures were reviewed and approved by the Institutional Animal Care and Use Committee at the University of California, Irvine, under protocol no. 2016–3199.

## III. RESULTS

**FIG. 4.**
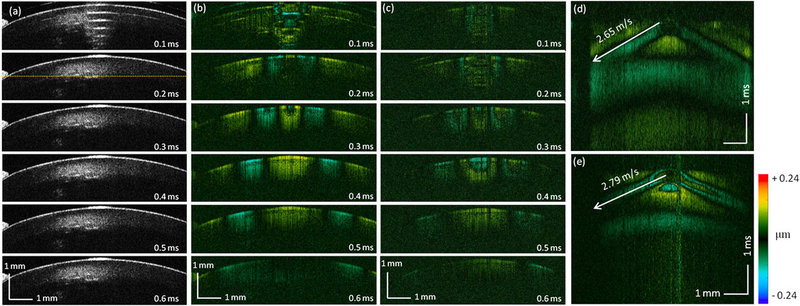
*Ex vivo* cold cataract lens results. (a) Time-lapse OCT B-scan images of a normal lens. (b) Time-lapse Doppler OCT B-scans of a normal lens. (c) Time-lapse Doppler OCT B-scans of a cold cataract lens. [(d) and (e)] Spatiotemporal Doppler OCT of the normal and cold cataract lens, respectively, at a depth indicated by yellow dashed line in (a).

An experiment based on a cold cataract model was performed *ex vivo* to test the proposed ARF-OCE system. Figure 4(a) shows the time-lapse OCT B-scan images of a rabbit crystalline lens. At 0.1 ms, fringe washout caused by acoustic radiation can be observed in the center of the OCT images, in which an elastic wave was induced. To visualize the wave propagation, the Doppler OCT images were obtained using (Eqs. 1) and (2) to display the localized axial displacement [Fig. 4(b)]. At 0.1 ms of Fig. 4(b), a significant displacement was observed at the center of the lens, where the focus of the ultrasound transducer was applied. Over time, the wave propagating bilaterally outwards was observed [Fig. 4(b)]. After the initial imaging, the lens was immersed in ice water to induce cataract. The corresponding Doppler OCT images are shown in Fig. 4(c). In order to quantify the Young’s modulus change, quantitative analysis was performed to visualize spatiotemporal Doppler OCT images of the lens at a selected depth [Figs. 4(d) and 4(e)], indicated by the yellow dashed line in Fig. 4(a). The elastic wave velocities were determined by calculating the ratio of travel distance to travel time, which were 2.65 m/s and 2.79 m/s in normal and cold cataract lens, respectively. The corresponding Young’s moduli were calculated to be 27.4 kPa and 30.4 kPa, respectively, using (Eq. 3). These results demonstrate the increased Young’s modulus of the lens in the cold cataract model, which was supported by a previous study.^40^

**FIG. 5.**
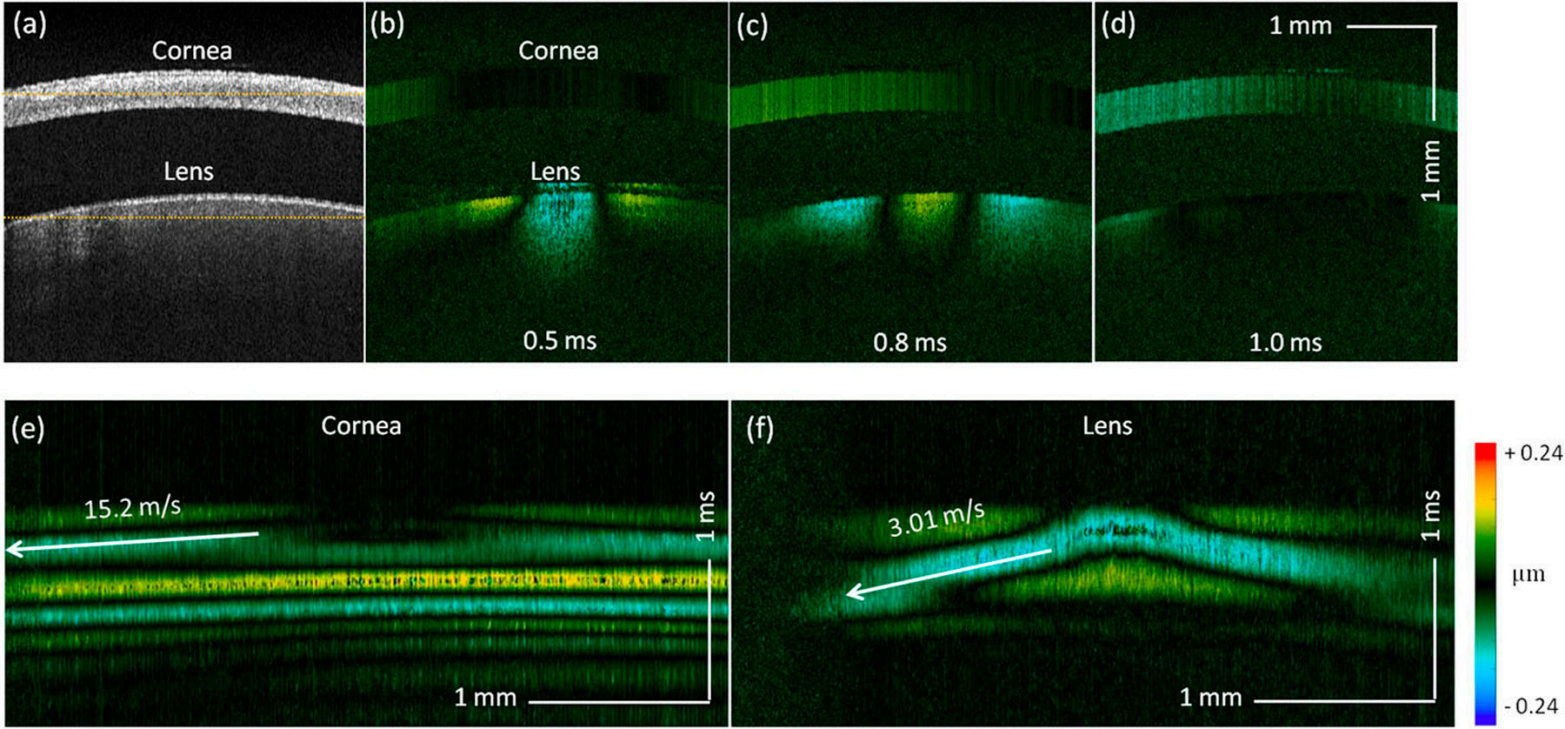
Simultaneous measurements of the lens and cornea in an *in vivo* rabbit model. (a) OCT B-scan images of the lens and the cornea. [(b)–(d)] Doppler OCT B-scans of lens and cornea at 0.1 ms, 0.4 ms, and 0.7 ms after ARF excitation, respectively. [(e) and (f)] Spatiotemporal Doppler OCT of the cornea and lens, respectively, at the depth indicated by yellow dashed lines in (a). Multimedia view: [(b)–(d)]: https://doi.org/10.1063/1.5118258.1

To study the elasticities of the lens and the cornea as a complete optical system, we performed an *in vivo* experiment to simultaneously measure the elastic waves of the lens and cornea in a rabbit model (~5 kg, n = 2). The ARF-OCE system has a sufficient imaging penetration depth to visualize both the cornea and the lens [Fig. 5(a)]. Figures 5(b)–5(d) (Multimedia view) demonstrate the propagations of the elastic waves in the cornea and the lens at different time points after a single ARF excitation. Figures 5(e) and 5(f) show the corresponding spatiotemporal images at two selected depths, indicated by yellow dashed lines shown in Fig. 5(a). The elastic wave velocity in cornea was measured to be 15.20 m/s in the proptosed eye, which was faster than the previously reported velocities of the *ex vivo* cornea.^48,49^ The elastic wave velocity in the lens was 3.01 m/s, which was similar to the velocity obtained from our *ex vivo* rabbit experiment.

**FIG. 6.**
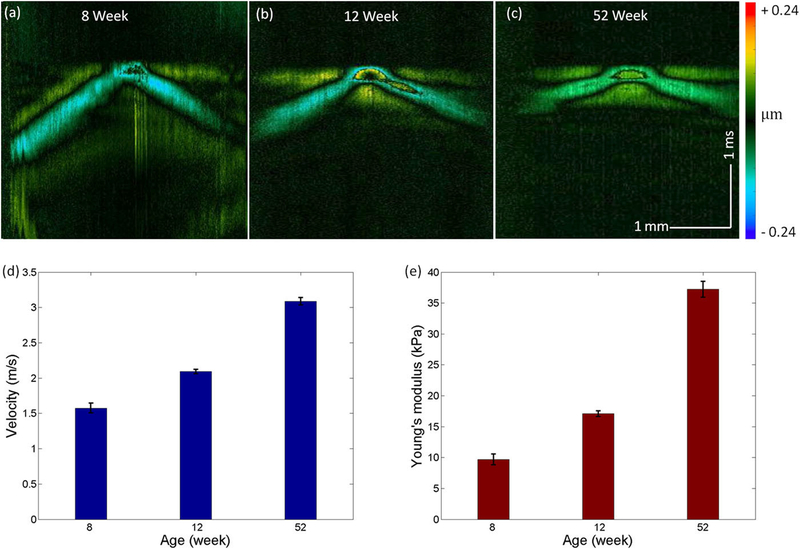
Results of the *in vivo* presbyopia rabbit experiment. [(a)–(c)] Spatiotemporal Doppler OCT of the lenses of the 8-week, 12-week, and 52-week rabbits, respectively. [(d) and (e)] Elastic wave velocities and Young’s moduli, respectively, of the corresponding ages.

Age-related loss of lens elasticity (i.e., presbyopia) is a common condition resulting in the decreased ability of the eye to focus on nearby objects. To further validate the ARF-OCE system, we performed *in vivo* elastography on a 2-kg (~8 weeks, n = 1), a 2.5-kg (~12 weeks, n = 1), and a 5-kg (~52 weeks, n = 1) male rabbit to evaluate the changes in lens elasticity with respect to aging. The spatiotemporal Doppler images of the three ages are shown in Figs. 6(a)–6(c). The youngest rabbit exhibited the slowest wave velocity, whereas the oldest one had the fastest. The measurements of the elastic wave velocity and Young’s modulus were repeated 5 times, and the means and standard deviations are shown in Figs. 6(d) and 6(e). A significant increase in lens stiffness with aging was observed, which is consistent with the reported studies.^22,50^

**FIG. 7.**
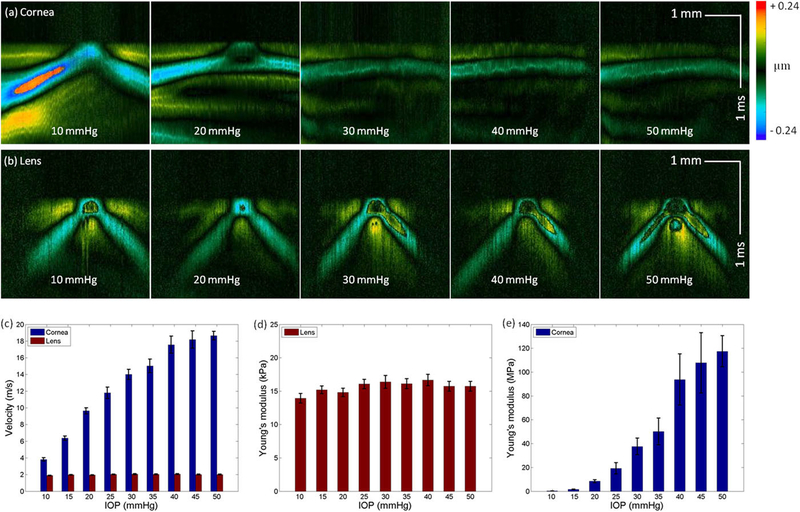
*In vivo* IOP experiments using a 12-week rabbit. [(a) and (b)] Spatiotemporal Doppler OCT of the cornea and lens, respectively, at different IOPs. (c) Elastic wave velocities in the cornea and lens. (d) Young’s modulus of the cornea as a function of IOP. (e) Young’s modulus of the lens as a function of IOP.

The positive correlation between corneal elasticity and IOP has been previously reported; here, we further investigated the change in lens elasticity with increasing IOP. A custom-built IOP monitoring and control system was integrated into the rabbit experiment, and ARF-OCE was performed at different IOPs. Figures 7(a) and 7(b) show the spatiotemporal cornea and lens Doppler images of the 12-week rabbit (n = 2) with different IOPs, respectively. Five measurements were taken from each rabbit, and the means and standard deviations were reported. Figures 7(c) and 7(d) demonstrate the increased wave velocity and Young’s modulus of the cornea with increasing IOPs. For the lens [Figs. 7(c) and 7(e)], although a minimal increase in the velocity and Young’s modulus is noted, the correlation between Young’s modulus and IOP is much weaker than that of cornea.

**FIG. 8.**
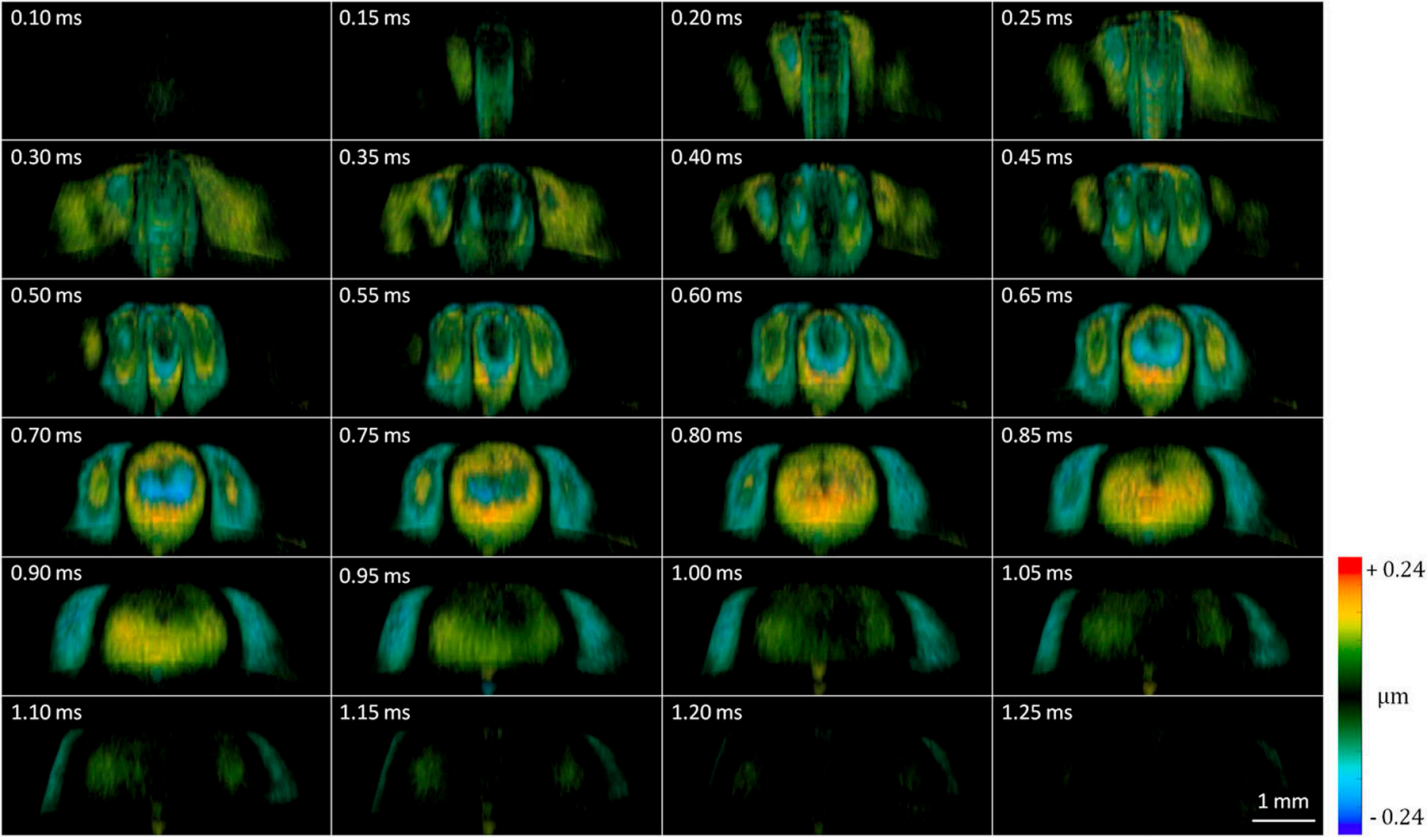
Time-lapse 3D Doppler OCT of the 12-week-old rabbit lens. Multimedia view: https://doi.org/10.1063/1.5118258.2

The lens is an inhomogeneous solid tissue with location-dependent stiffness. Three-dimensional (3D) visualization of elastic wave propagation will allow for accurate characterization of the lens biomechanical capacity and heterogeneity. Figure 8 (Multimedia view) shows representative time-lapse 3D Doppler images. ARF excitation was employed at 0.15 ms, indicated by fringe washout at the center of the field of view. The outward propagation of the spherical wave traveling at an average speed of 3 m/s was observed.

## IV. DISCUSSION AND CONCLUSION

The crystalline lens and the cornea as a complete optical system are the crucial elements in human vision. Understanding the biomechanical capacity that facilitates the eye accommodation process to focus near and far objects will advance not only our knowledge in vision science but also in developing better clinical management for relevant ocular diseases. Previous *in vivo* studies of anterior eye elasticity focused on the easily accessible cornea, but *in vivo* lens elasticity studies remain challenging. Our ARF-OCE system, which incorporated a swept-source light with oblique illumination, provides improved performance and thus enables real-time simultaneous *in vivo* measurements of the lenticular and corneal Young’s moduli.

We first tested the ARF-OCE system through the *ex vivo* cold cataract model, in which the increase in lens elasticity was detected. Through the *in vivo* rabbit model, we demonstrated the system capability to simultaneously quantify lens and cornea elasticity, and finally, the dependencies of age and IOP with respect to lens elasticity were investigated. We confirmed the age-related lens hardening, which was consistent with previous work.^50^ We also identified an overall positive trend of the lens elasticity in the IOP experiment, but the influence on lens elasticity from changes in IOP was insignificant compared with the cornea. Lens experiencing weak deformation with increasing IOP compared to other ocular tissue, such as cornea and sclera, has been previously observed.^38^

While the proposed ARF-OCE system is a promising *in vivo* imaging method for quantifying anterior eye elasticity, a few challenges still need to be addressed in order to successfully translate this technology for clinical applications. First, a faster imaging speed is necessary to minimize artifacts induced by bulk motion of the body, especially for animal models whose respiratory and heart rates are much higher than that of a human. Additionally, high-speed imaging will enable 3D volumetric imaging of the anterior eye with a single-shot ARF excitation, which will reduce the procedure time, hence minimizing the light and ARF exposures. Recently, Fourier domain mode locking (FDML) lasers providing laser repetition rates in the megahertz range have been commercialized and can be utilized to improve the imaging speed. Additionally, the current imaging protocol for the rabbit model in our experiment utilizes orthogonal ARF excitation that requires proptosis of the eye, which increases the IOP up to 50 mmHg and affects the OCE results. Ideally, a normal IOP (ranging from 12 to 22 mm Hg) should be maintained during elastography measurement. Although this can be achieved by our IOP controller system, it increases the procedure invasiveness and risks and is clinically impractical. An alternative method is a fluid bath, which is clinically used in anterior segment ultrasound to couple the transducer; this may eliminate the required eye proptosis and better facilitate clinical translation. Furthermore, the mechanical index (MI) of the applied ARF in our study is approximately1.5, which is much higher than the FDA ophthalmic MI standard of 0.23.^51^ In our experiment, the displacement induced by the ARF is hundreds of nanometers, while the phase sensitivity of our system is less than 1 nm. Therefore, decreasing the excitation force by 10-fold is feasible to achieve safety MI. Our future study will focus on determining the minimal excitation force while maintaining adequate mechanical index. Finally, elasticity quantification in 3D is essential for the heterogeneous tissue. In this study, we have presented the visualization of elastic wave propagation in 3D to demonstrate the feasibility of 3D quantification of elasticity. In the future, more ocular tissues will be tested to provide a 3D quantitative elasticity mapping.

In summary, we have reported the first *in vivo* lens elasticity measurement. Additionally, the ARF-OCE system allows for mapping and quantification of the mechanical properties of lens and cornea simultaneously, and our technique has been validated in an *in vivo* rabbit model. Ophthalmic anterior segment elastography enabled by the proposed OCE system is able to identify the important biomechanical parameters that have great potential to help advance the vision science research and improve clinical management of patients with refractive vision disorders.

## Supplementary Material

Video for Figure 5

Video for Figure 8
